# Report of Cases

**Published:** 1828-01

**Authors:** Alexander Rainy

**Affiliations:** *the* Aberdeen New Dispensary senior Pupil.


*Report of Cases treated at the* ABERDEEN NEW DISPENSARY *and communicated by* ALEXANDER RAINY, senior Pupil.

(Continued from vol. iii. p. 487.)

VI. INJURY OF THE SPINE.

March 11th, 1827.—James C—, ætatis forty-eight. Found this patient supported in a chair, and in an insensible state, with his chin resting on his breast, unless when raised by assistants. He was pale, and his pulse at the wrist was not to be distinguished, nor could the heart's motion be felt through the thoracic parietes. While unloading a cart a few minutes previously, a heavy cask had fallen on his breast, by which he was driven to the ground, his head against the wall, and the cask above him. Stimulants were given, and, when reaction appeared, a vein was opened, from which the blood flowed at first drop by drop; he was then able to speak a little, but not reply to questions. Pulse now sixty, very weak. On examining the body very carefully, it was found that the vertebra dentata could be more distinctly felt than in the natural state, while the atlas seemed to have receded, and the muscles of the part were on the stretch, notwithstanding the head was thrown back during this examination. The head, left to itself, fell forwards till the chin rested on the chest, and, as the insensibility disappeared, it was irregularly and spasmodically darted forwards. The legs and arms were completely paralysed, being pinched or pulled with any violence without the least sensation of uneasiness. The man was conveyed home; a door was laid on his bed, sloping from the top to the bottom; over this an even mattress was placed, and he was extended on it, while the head was drawn with some firmness in the line of the vertebræ. After this, the muscles on the stretch relaxed, and the dentata seemed less projecting, while the pulse became fuller and stronger, though of the same frequency as before; and he expressed a desire to evacuate the contents of his bowels. The head was secured by tapes in the proper posture, and he was left for the night.

12th.—Had a natural stool last night, and urine this morning; both after some effort. Body under the neck somewhat colder than the head, which appears flushed. Muscles still insensible in the extremities; slight sensation in the muscles of the chest and abdomen. Slept none all night. Pulse oppressed; the same frequency. Was bled again to fifteen ounces, during which the pulse rose in strength. At the end of the operation it began to get weaker, and was stopped. The prick of the lancet was not felt, through the lower jaw was convulsively drawn up when the wound was made, or when touched during the operation.

> Friction with the flesh-brush and mustard flour, for an hour at a time, twice a day.—Three ounces of black draught.

Seven P.M.—Purgative has just operated. Felt some stinging in the arms and legs during the application of the brush. Face still flushed, but no headache.

14th.—Yesterday had severe pains in the bowels, which were a good deal distended with flatus. Ordered fomentation of chamomile, which eased the pain; and an enema, which was not, however, exhibited. Towards evening, severe retching, unrestrained by a cardiac mixture, and only abated when he turned to his side, in which posture he was supported some hours. Bowels still much distended. Has had a discharge of urine twice today, but complains much of the efforts necessary to void his fæces, which are free. Enema ordered. Frictions have been discontinued today.

16th.—Friction resumed yesterday. Has some feeling in the right arm and left leg; knows when a cold hand is laid on them, or the skin pinched. Yesterday morning, after a tolerable night's rest, was waked by severe pains in his lower extremities, which continued till the visit in the afternoon, when they were eased by substituting the anodyne liniment for the mustard. Today, occasional darting pains through his legs; once or twice his right leg was involuntarily drawn up with considerable force and excruciating pain. In lying, he bends the right thigh a little towards the body, and can maintain it in that posture for a few minutes. The anodyne at night invariably produces vomiting, and consequently left off. Bowels loose yesterday; one natural stool today. Can pass his water with great ease, but still complains of the exertion at stool. Pulse seventy-two, full and soft; tongue dry and clammy; occasional nausea. Ordered the saline effervescing draughts. Bowels distended with flatus. Beer, which he drinks in considerable quantity, to be denied him.

As the details of this case are given at considerable length, we are compelled to give a very abridged account of what remains. As the frictions seemed to be very beneficial, they were not discontinued till a few weeks ago. Blisters were applied along the spine in succession, and kept discharging as long as they could be borne. The remainder of the treatment was confined to regulation of digestive organs. The feeling and power of motion were restored to the lower extremities two months after the accident, so far as to allow his walking across the room, with as much assistance as was required to hinder him from falling backwards, which he would readily have done if left to himself. The right arm is so far restored as to enable him to take food with that hand, but the left remains in a very powerless state. He now walks about the city for many hours daily, but is unable to apply himself to any employment, from the unsteadiness of the muscles of the back and legs. It is proposed to try the effect of electricity on the left arm.

We are afraid to hazard any opinion on the above case: it seems evident that the ligaments connecting the atlas to the dentata were ruptured, and it is only surprising that life was preserved. Notwithstanding two months of close confinement to bed, the patient's health at that time was good.

### VII. GLOSSITIS.

Case I. June 27th.—John Ogilvy, æt. fifty-five, has swelling of tongue to such an extent as to prevent deglutition and speech entirely; cannot put the tip of the tongue over the incisor teeth. Complaint of two days' standing. Has the charge of a steam-engine, and exposed to alternations of heat and cold. Had a rigor on leaving work three days since, and has been always sick on going out during that time. Constant dribbling of saliva from the mouth; bowels costive; pulse seventy-eight, weak.

Sixteen leeches applied to the jaws. Purgative enema.

30th.—Relieved in a slight degree by the leeches. Can articulate so as to be understood. Bowels opened by the enema.

Repeat the leeches. Apply blisters behind the ears; dress with savine ointment. Let him endeavour to swallow three and a half ounces of black draught.

July 1st.—Bowels well opened; speech freer; cannot put out the tongue. Blisters discharged well.

2d.—Tongue considerably reduced, but cannot be put out farther than the lips; marked over its margin with pits, corresponding with the teeth opposite to which it lay when much swelled, and into the interstices of which it had forced itself. Bowels slow.

Repet. Haust.

6th.—Tongue much reduced, though his speech is yet a little thick and confused. Dismissed.

As glossitis occurring as an idiopathic disease is rather unusual, it will not be deemed improper to go back a few months for the following case:

CASE II. January 31st.—Mrs. Conolly, æt. thirty-two, married, but childless, caught a severe cold three days ago, when she says the throat was much swelled, with stiffness at the root of the tongue: deglutition of fluids being impossible, on account of going, as she described, "into the wrong passage," and bringing on severe cough. This increased till the whole tongue became swollen and stiff. She cannot now bring the teeth close, on account of the size of the tongue, which fills the mouth. Continual, and very troublesome, running of saliva from the mouth. Cannot articulate so as to be understood, farther than yes or no. Face is somewhat flushed; pulse 100, full and hard; bowels costive; no sleep for two days.

V.S. ad ℥xvij. Cathartic enema. Feet to be bathed in warm water; and a dozen of leeches to the angle of the jaw.

February 1st.—Swelling as yesterday; patient extremely fretful and impatient. Enema brought away some hard fæces. Saliva dribbling away in greater quantity than before. Makes signs that the neck feels stiff and sore; pulse ninety-two, soft.

Repeat the leeches and enema, and, after the bleeding, apply blisters along both sides of the lower jaw, as far as the chin, and dress with savine ointment. Inhale the steam of warm water, if possible, by the mouth.

2d.—Can close the mouth, and articulates so as to be understood with some difficulty. On these occasions the saliva runs from the mouth. To endeavour to swallow some of the Mist. Purg. today, as the enema brought nothing last night.

4th.—Complains bitterly of the pain of the blisters, which are discharging freely. Voice still somewhat indistinct; tongue can now be seen, and is of a red shining appearance. Purgative, which she swallowed yesterday, has produced several copious dark stools. Repeat the cathartic.

After this, she remained for a fortnight with inflammation of the right tonsil, which, in spite of the active measures employed, went on to suppuration; the digestive organs becoming seriously deranged, with bilious vomiting.

### VIII. SUDDEN DEATH.

August 1st, 1827.—James Mathews, æt. forty-eight: cough, with purulent expectoration; lies with difficulty on the right side; rest disturbed by startings and disagreeable dreams; heart palpitates on using any active exertion; pulsations regular. Dyspnœa occurs in paroxysms; health otherwise good. Cough of several years' duration, being present in the winter season only. This attack has continued from November last year; says he found Squill mixture of most advantage in his former attacks.

Ordered Compound Powder of Jalap at bedtime.

2d.—Chest examined today with considerable care: slight mucous rattle could be heard by the unassisted ear, and more distinctly by the stethoscope; percussion elicited the usual sound of the healthy chest. Some fulness in the epigastrium and right hypochondrium. Cough as yesterday; obscure sense of fulness at the left side, under the false ribs. Made him walk smartly through the room for some minutes, but no palpitation occurred. One costive stool from the powder.

Repeat the powder.

3d.—Last night had a severe fit of coughing, followed by smart pains through the chest and bowels; to relieve which, he insisted on his attendants procuring him an enema. After the injection, he had a call to stool: while rising, he fell into his nurse's arms, and expired.

5th. *Sectio cadaveris.*—Chest laid open: cartilages of the ribs almost entirely ossified. Lungs apparently healthy: on being cut into, they were found gorged with blood. Some white frothy mucus in the bronchiæ. A few tubercles, of the size of a pea, were found through the substance of the different lobes. The pericardium contained an ounce and a half of clear serum. The heart healthy every-where, except some round cartilaginous bodies, of about two lines in thickness, in the mitral valve. Spleen enlarged, and distended with fluid blood. Liver hard, pale, and extending across the epigastrium. Stomach and bowels sound; mucous surface of the stomach had a slight blush of redness.

*Remarks.*—As anticipated from the indications of the stethoscope, no enlarged tubercles or ulcerations of the lungs were found after death. We presume that the cartilaginous degeneration of the mitral valve was sufficient to account for the palpitation and disturbance during sleep. As Mathews had been for some years in the West Indies, and with our army in Egypt, it is probable that he there acquired the liver complaint, which he never mentioned during his life, he being particularly taciturn.

Query, were the granular bodies in the valves the cause of this man's death?

### IX. VACCINIA.

The following are curious cases, forming exceptions to some of the best established laws of morbid phenomena.

Case I. June 7th.—Widow Aird, æt. forty, the mother of Charlotte Aird, at present ill of confluent small-pox, attended the

Dispensary five or six days since, and states that she sat for a few minutes beside a child who had been vaccinated seven days previously, and from whose arm matter was taken to vaccinate other children. She states, that on the following day she had severe rigors, nausea, lassitude, and pains of the back and head, which she attributed to the fatigue of attending on her daughter. This was the more probable as she had had the small-pox in her childhood. Three days ago, however, the pyrexia having abated, she observed a small pimple on the forearm of the left side, which put on the appearance of the vaccine vesicle. Today it shows all the appearances of this last, having the depression in the centre and the usual surrounding areola of inflammation. Two others have come out on the legs, of the same character: one, however, less distinct, the vesicle being torn and inflamed.

On the 11th, Catherine Aird, a child of three years, was attacked with chilliness and pains in the head and bowels. On the 12th, she was feverish, and vomited.

14th.—Symptoms were today alleviated by an emetic and cathartic.

15th.—A vaccine vesicle has made its appearance, having the depression in the centre, circular, filled with clear lymph, and with the surrounding erythema in a slight degree. This was first noticed by the mother on the afternoon of the 12th. On examining the body, another vesicle was found on the thigh, in a less advanced state, and a third on the cheek, not yet risen into a blister. Skin is now cool, and the head easy.

16th.—Last night the fever again came on, when several small pimples appeared, which show today all the marks of regular small-pox, rather as modified by vaccination.

17th.—Some of the pustules are filled with pus; they are pretty numerous on the face, arms, and breast. Fever again abated.

21st.—Pimples have reached the usual size, and are numerous on all parts of the body. No fever.

23d.—Pustules on the face have scabbed.

*Remarks*.—We have given the above cases as found in the Report-book, omitting the treatment and remarks; vouching for the correctness of the statements.*

### X. PHTHISIS, WITH HEPATISED SPLEEN.

July 1st, 1827.—Jane Myres, æt. twenty-three, has been confined to her bed for the last eighteen months, but gives a very imperfect account of the origin of her complaint. When we saw her, six weeks since, for the first time, she laboured under cough,

* These appear to us to be examples of the mild form of varioloid disease which sometimes arises from exposure to the contagion of small-pox; and, so far as they go, they tend to prove the identity of all those diseases.—E.

with purulent expectoration, diarrhœa, and irritability of stomach, rejecting most kinds of food; tongue pretty clean and moist; skin moist; had not been troubled with night sweats since the occurrence of the diarrhœa, which was particularly troublesome. Anodyne clysters moderated the looseness from that time till her death, two days since. Attention was principally directed to the stomach, which was so irritable at last that even the mildest ripe fruits were rejected. No pain was evinced on pressure, and the tongue was nearly in the natural state. All remedies tried for the relief of this symptom were only of temporary benefit, and no other nourishment was taken for the last fortnight of her life but what was contained in clysters made with arrow-root, in place of the starch in the anodyne injection.

*Appearances on dissection.*—On opening the thorax, the pleura costalis and pulmonalis were connected by strong adhesions. A considerable quantity of coagulable lymph adhered to the surface of the lungs on the left side. On separating the strongest of these, we found, a little way in the substance of the middle lobe, an irregular cavity of about four inches, greatest length, lined by a firm fibrous membrane of considerable thickness. One or two small imposthumes were found in the right side.

The abdomen was then opened, when the parts presenting appeared healthy, excepting the spleen, which was enormously enlarged, and weighed four pounds. On being cut into, it presented the appearance of the sound liver, but considerably firmer; its vessels were enlarged, but no blood could be pressed from it. It occupied the whole of the left hypochondrium, and the stomach was found pressed nearly into the centre of the epigastrium.

### XI. EXTRAVASATION ON THE BRAIN.

May 25th, 1827.—Margaret Pillendugh, æt. thirty-eight, of a sanguineous temperament, was passing through a narrow alley where a house was building, when a stone was supposed to have been thrown by the workmen from the top, which struck her on the crown of the head.

Half-past five P.M.—Found her in bed, in an insensible state, with a small wound on the scalp, crossing the sagittal suture at its back part, without any injury of the parietal bones. The wound had bled freely. After dressing it, the other symptoms were inquired into: the breathing was oppressed and stertorous, the pupil in a semi-contracted and fixed state; pulse sixty, oppressed; the jaws were locked, but could be separated a small way; the lower jaw drawn in under the upper. On the arms being pricked, she drew them under the bed-clothes; attempted once or twice to cough, seemingly to relieve herself from mucus in the trachea; and slight convulsive twitches occurred, during one of which the under lip was severely bit. The contents of the stomach were worked out from the mouth, frothing around the lips, and sometimes blown out with violence, but without separating the teeth; coldness in the lower extremities.

Brandy-and-water in small quantities till retching take place; and a stimulating injection immediately.

Ten P.M.—Injection brought off some solid fæces; urine passed in bed; insensibility still continues; pulse seventy-two, with some firmness. Was bled to twelve ounces, during which the pulse rose in frequency, but fell soon after.

26th, half-past ten A.M—Coma continues. Much starting of the tendons, particularly of the left arm. Left corner of the mouth drawn upwards; pupils dilated; strong grinding of the teeth; snores loudly, with intervals of placid breathing; skin moist; pulse seventy-two, again somewhat firm.

V.S. ad ℥xij.

Eight P.M.—Blood cupped, first cup buffy; redness of the cheeks considerable; heat and moisture over the body; mouth not drawn up as in the morning; subsultus tendinum more frequent; no action in the wound.

Repeat the bleeding and enema. Keep the head wet.

27th, ten A.M.—Symptoms much as yesterday. Pulse eighty-four, firm and throbbing; flushing of the face gone. Blood to be abstracted from the temples or jugular vein, till the throbbing of the pulse is removed. Blood drawn in the morning cupped and buffy.

Eight P.M.—About eight ounces of blood was drawn from the temporal artery, when the pulse at the wrist suddenly sunk, so as to be scarcely felt. Bleeding was then discontinued, and the brandy-and-water given till the pulse returned. This was at three P.M. At the visit, the pulse was again eighty-four, bounding; the countenance flushed, with drops of perspiration on the face; breath free from stertor. Effusion beginning to take place into the bronchiæ.

28th.—Died this morning, at five o'clock.

*Sectio cadaveris.*—The usual incision from ear to ear being made, we proceeded to turn down the scalp, when we found under the posterior fold a large quantity of effused blood, more abundant on the right than on the left side of the brain, and greater as it approached the base of the skull. No fracture or fissure could be discovered in this stage of the dissection. Proceeding to remove the skull-cap, the whole of the posterior part of the dura mater was found covered with coagula, which were in greatest quantity on the right side. Under this membrane, on the surface of the cerebrum, blood was effused in considerable quantity. Brain and cerebellum removed; texture natural; lateral sinuses full of dark blood. A clot in the longitudinal sinus, anterior to its bifurcation, crossing the horizontal limb of the cruciform spine of the occipital bone. A fine fissure was discovered, forming two-thirds or more of a circle, which the saw (in removing the skull-cap) has completed. This was of the size of a large walnut, and from the lower boundary a crack extended down to the edge of the foramen magnum, but not through it. There was no depression, nor could it be pressed inwards, but yielded readily to pressure from within. No diseased appearance in the brain or its membranes.

### XII. MELANOSIS.

June 31st, 1827.—James Cow, æt. six, had been long affected with mesenteric disease previous to his death, which took place two days since. The symptoms were entirely those peculiar to that disease. For the last twelve months, hectic sweats and diarrhœa alternated, and, for the last few weeks of his life, he suffered severely from sharp pains darting through his belly in different directions.

On opening the body, adhesions were found between the different folds of the intestines. Some of the mesenteric glands were as large as a nut, and hard. The peritoneum lining the abdominal muscles, and that covering the intestines, was studded over with small, raised, and black dots. They were found also upon the surface of the liver and stomach; broadest, however, upon the first-mentioned part, where the largest was nearly the size of a pea. None of these were found in the other cavities.

This child passed considerable quantities of black blood by stool and urine, for a few days before his death.

*Aberdeen; 28th August*, 1827.

